# Autophagy-Related Atg8 Localizes to the Apicoplast of the Human Malaria Parasite *Plasmodium falciparum*

**DOI:** 10.1371/journal.pone.0042977

**Published:** 2012-08-10

**Authors:** Kei Kitamura, Chieko Kishi-Itakura, Takafumi Tsuboi, Shigeharu Sato, Kiyoshi Kita, Nobuo Ohta, Noboru Mizushima

**Affiliations:** 1 Department of Physiology and Cell Biology, Tokyo Medical and Dental University, Tokyo, Japan; 2 Department of Environmental Parasitology, Tokyo Medical and Dental University, Tokyo, Japan; 3 Cell-Free Science and Technology Research Center and Venture Business Laboratory, Ehime University, Matsuyama, Ehime, Japan; 4 Division of Parasitology, MRC National Institute for Medical Research, London, United Kingdom; 5 Department of Biomedical Chemistry, Graduate School of Medicine, The University of Tokyo, Tokyo, Japan; Bernhard Nocht Institute for Tropical Medicine, Germany

## Abstract

Autophagy is a membrane-mediated degradation process, which is governed by sequential functions of Atg proteins. Although Atg proteins are highly conserved in eukaryotes, protozoa possess only a partial set of Atg proteins. Nonetheless, almost all protozoa have the complete factors belonging to the Atg8 conjugation system, namely, Atg3, Atg4, Atg7, and Atg8. Here, we report the biochemical properties and subcellular localization of the Atg8 protein of the human malaria parasite *Plasmodium falciparum* (PfAtg8). PfAtg8 is expressed during intra-erythrocytic development and associates with membranes likely as a lipid-conjugated form. Fluorescence microscopy and immunoelectron microscopy show that PfAtg8 localizes to the apicoplast, a four membrane-bound non-photosynthetic plastid. Autophagosome-like structures are not observed in the erythrocytic stages. These data suggest that, although *Plasmodium* parasites have lost most Atg proteins during evolution, they use the Atg8 conjugation system for the unique organelle, the apicoplast.

## Introduction

**Figure 1:**
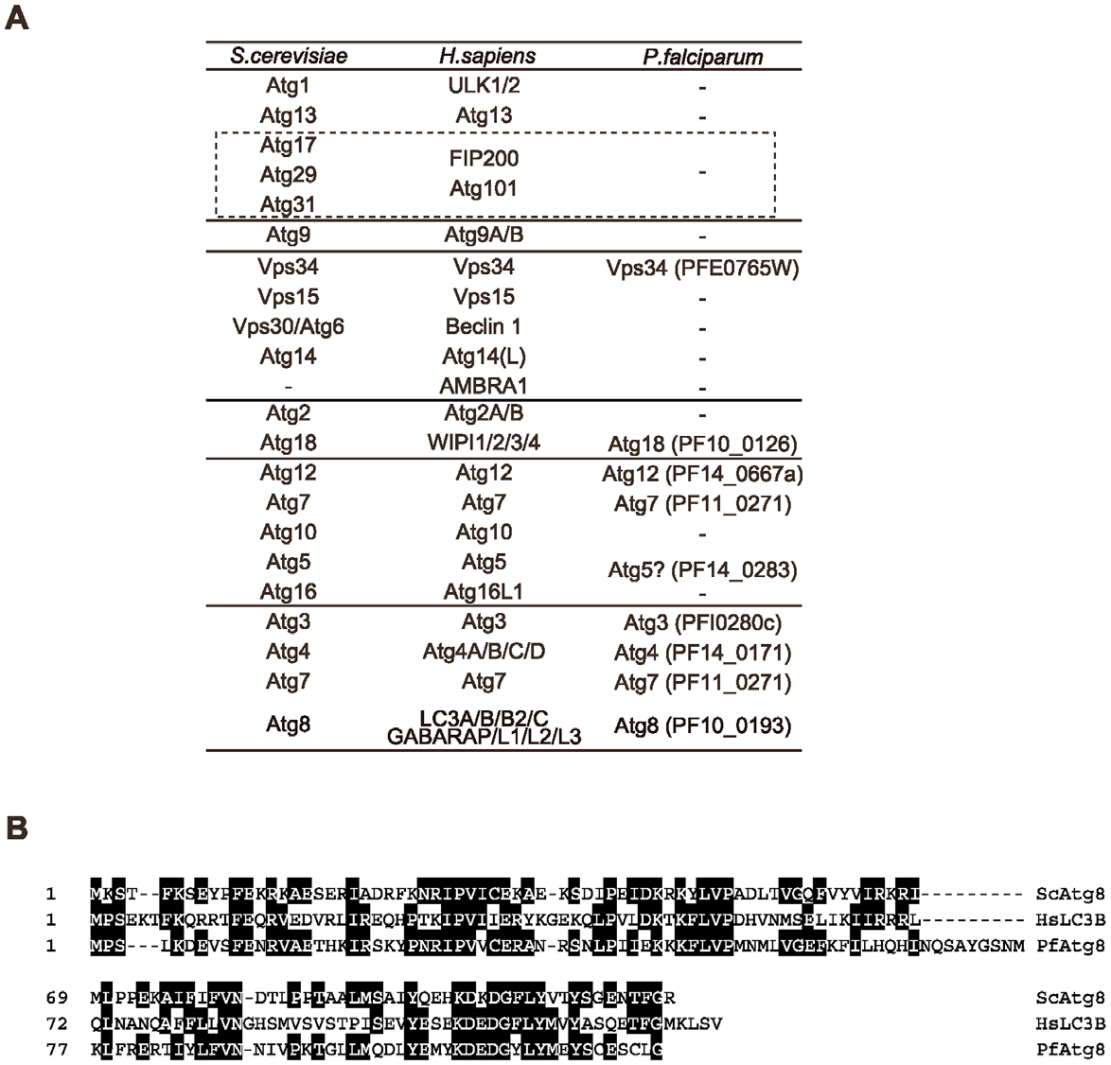
Atg protein sets are only partially conserved in *P. falciparum*. (A) List of Atg proteins in *S. cerevisiae*, *Homo sapiens* and *P. falciparum*. –, no ortholog found. It has been suggested that the mammalian FIP200–Atg101 complex and the yeast Atg17–29–31 complex are functional counterparts (dashed boxed) although they do not show significant sequence similarities. None of these factors seems to be conserved in *P. falciparum*. The tag of locus in the *P. falciparum* genome is indicated in parentheses. (B) Alignment of the full sequences of *S. cerevisiae* Atg8, *H. sapiens* LC3B (one of the Atg8 homologs), and *P. falciparum* Atg8. Identical amino acid residues are indicated with filled boxes.

Macroautophagy (simply referred to as autophagy hereafter) is a fundamental cellular process, by which cytoplasmic components including proteins and organelles are delivered to the lysosome (or vacuole in yeasts and plants) for degradation. Autophagy is involved in many cellular functions such as adaptation to starvation, cell differentiation, quality control of proteins and organelles, aging, and degradation of invading microbes [1], [2], [3], [4], [5], [6]. It is also implicated in human diseases such as cancer, inflammatory diseases, and neurodegeneration. Autophagy involves complex membrane dynamics; a membrane cisterna termed the isolation membrane (or phagophore) elongates on the endoplasmic reticulum (ER) and forms a double membrane-bound autophagosome, which contains cytoplasmic materials. Then, the autophagosome fuses with a lysosome to degrade the enclosed materials. Autophagosome formation is the central event of this process and is governed by autophagy-related (Atg) proteins, which were originally identified in yeast [7], [8]. The genetic hierarchy of these Atg proteins has been determined and they are classified into at least six functional groups: the starvation-responsive Atg1 kinase complex (Atg1–Atg13–Atg17–Atg29–Atg31), the multi-membrane spanning protein Atg9, the class III phosphatidylinositol 3 (PtdIns 3)-kinase complex (Atg6–Atg14–Vps15–Vps34), the Atg2–Atg18 complex, the Atg12 – Atg5–Atg16 complex (“–” denotes a covalent attachment), and the Atg8–phosphatidylethanolamine (PE) conjugate (Figure 1A) [8], [9], [10].

These core Atg proteins are highly conserved in most eukaryotes including fungi, animals, and plants [11]. However, recent genome-wide analyses have revealed that they are only partially present in protozoa [12], [13]. It is interesting that their conservation pattern is not random; the members belonging to the Atg8 conjugation systems are highly conserved in almost all protozoans, whereas potential homologs of other Atg proteins are only found sporadically (Figure 1A) [12]. The ubiquitin-like protein Atg8 can be covalently conjugated to PE through a sequential reaction that is mediated by a ubiquitin E1-like enzyme, Atg7, and an E2-like enzyme Atg3 [14]. Atg4 cleaves the C-terminal extension of the proform of Atg8 to expose a glycine residue, to which PE is conjugated. Atg4 also catalyzes deconjugation of the PE moiety from Atg8 – PE to release Atg8 from the membrane after completion of autophagosome formation [15]. Although the precise function of Atg8 and its PE conjugation in autophagy remains unclear, it is suggested that Atg8 – PE is important for membrane tethering and hemifusion [16], determination of the autophagosome size [17], and expansion and closure of the isolation membrane [18], [19], [20]. The partial conservation of the *ATG* genes in protozoans might imply that the smaller set of Atg proteins is sufficient to constitute the autophagosome in these organisms. Alternatively, these organisms may use the Atg8 system for other purposes.

To date, several functional and morphological analyses of autophagy have been performed in protozoan parasites [13]. *Entamoeba invadens* possesses the Atg8 system, but lacks the Atg12 system. Atg8-positive vacuolar structures are generated in a PtdIns 3-kinase-dependent manner during encystation, but its ultrastructure is unknown [21]. In *Trypanosoma cruzi*, autophagosome-like double-membrane structures are formed in epimastigotes and implicated in differentiation into metacyclic trypomastigotes [22], [23]. *Leishmania major* seems to have both Atg8 and Atg12 systems [24], and Atg8-positive punctate structures are observed during metacyclogenesis [25]. Accordingly, Atg4-deficient *L. major* shows a defect in differentiation into metacyclic promastigotes [25]. A more recent study performed in *Toxoplasma gondii* showed that genetic depletion of *TgAtg3*, which encodes an enzyme required for Atg8 – PE conjugation, causes growth inhibition and mitochondrial anomalies, which may be due to a defect in mitophagy [26].

In contrast, the nature of Atg proteins of the malaria parasite *Plasmodium* spp. remains largely unknown. *Plasmodium*, which belongs to phylum Apicomplexa together with *Toxoplasma*, possesses characteristic organelles such as the apicoplast, rhoptry, microneme, and dense granule. The *Plasmodium* sporozoite is transmitted by mosquito and first infects the hepatocyte which generates a large number of infectious merozoites. The merozoite infects erythrocytes and multiplies by schizogony to generate up to ∼32 merozoites. Finally the infected erythrocytes rupture, and newly formed merozoites are released into the blood stream. An electron microscopy study of the rodent malaria parasite *P. berghei* demonstrated the presence of autophagosome-like double-membrane structures, which appeared to eliminate micronemes in liver-stage parasites [13], [27]. Furthermore, *P. berghei* Atg8 appears to localize to abundant vesicles organized in a reticular network [27].

Because Atg8 – PE is present on both elongating isolation membranes and complete autophagosomes [28], [29], Atg8 and its orthologs have been generally recognized as an autophagosome marker. Thus, in this study, we determined the biochemical properties and subcellular localization of Atg8 in *P. falciparum*, the major cause of human malaria. Contrary to our expectation, we found that *P. falciparum* Atg8 (PfAtg8) was specifically associated with the apicoplast, not autophagosomes, during the erythrocytic stage.

## Results

### Expression of PfAtg8 increases during the erythrocytic stage

Previously, it was reported that the *P. falciparum* genome has only a partial set of core Atg proteins [12], [13]. We systematically searched for the orthologs of Atg proteins in the parasite genome and reached a similar conclusion (Figure 1A). We found genes encoding orthologs of a complete set of the Atg8 conjugation system (Atg3, Atg4, Atg7, and Atg8), although other *Atg* genes are only partially conserved (Figure 1A). Compared with Atg8 of the yeast *Saccharomyces cerevisiae*, PfAtg8 shows approximately 40% identity and 65% similarity and has the exposed C-terminal glycine residue, unlike Atg8 orthologs of other organisms (Figure 1B). *P. falciparum* possesses the class III PtdIns 3-kinase Vps34 [30]. In addition, although similarity is not high, there seem to be potential homologs of Atg5 (Figure S1), Atg12 (Figure S2), and Atg18 (Figure S3). Proteins encoded by *PF13_0116* and *PF14_0294* are partially similar to Atg2 and Vps15, respectively, but it remains unknown whether they are orthologs of Atg2 and Vps15.

**Figure 2:**
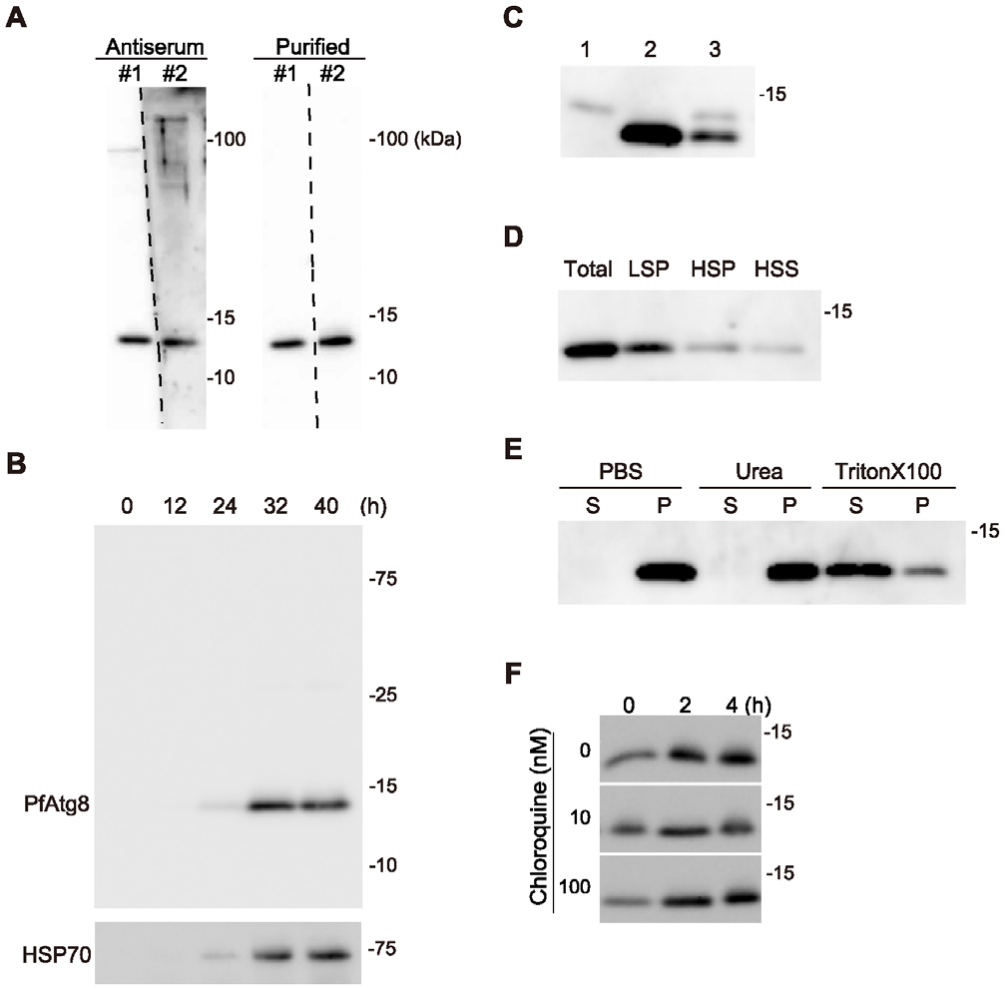
PfAtg8 is associated with membranes. (A) Specificity of the two independently generated anti-PfAtg8 antibodies (#1 and #2). Crude antisera and purified antibodies were used for immunoblotting of lysates of asynchronized *P. falciparum* parasites. (B) Expression of PfAtg8 increases during the erythrocytic stage of development. Highly synchronized *P. falciparum* parasites were collected at 0, 12, 24, 32, and 40 h after invasion. The duration of one cycle of the erythrocyte stage was approximately 42 h. Expression levels of PfAtg8 were analyzed by immunoblotting. An antibody against HSP70 was used as a loading control. (C) PfAtg8 exogenously expressed in mammalian cells (lane 1), endogenous PfAtg8 expressed in *P. falciparum* (lane 2), and the mixture of these two samples were subjected to immunoblot analysis using anti-PfAtg8 antibody. (D) Lysates of asynchronized *Plasmodium* were separated into low-speed (13,000×*g*) pellet (LSP), high-speed (100,000×*g*) pellet (HSP), and high-speed supernatant (HSS) fractions, and analyzed by immunoblotting using anti-PfAtg8 antibody. (E) The LSP fraction prepared in (D) was treated with 2 M urea or 2% Triton-X 100 and separated into 100,000×*g* pellet (P) and supernatant (S). (F) Infected erythrocytes were cultured in the presence of the indicated concentration of chloroquine and expression of PfAtg8 was analyzed.

To analyze endogenous PfAtg8, we generated two independent rabbit polyclonal antibodies against PfAtg8. Both antibodies specifically reacted with a band at approximately 14 kDa (Figure 2A). This size was close to that of Atg8 proteins of other species [28], [29]. We used the anti-PfAtg8 antibody #1 in the following experiments unless otherwise specified. The expression level of PfAtg8 was low during early intra-erythrocytic development, but it increased as the parasite matured and reached the maximal level at the late schizont stage (Figure 2B). This pattern was similar to that of HSP70 serving as a cytosolic loading control, suggesting that the apparent increase of PfAtg8 corresponds to the increasing volume of the intra-erythrocytic parasites.

### PfAtg8 is associated with membranes

In other organisms, Atg8 is present in two forms: free Atg8 and the membrane-associated form that conjugates with PE embedded in the lipid bilayer. The PE-conjugated and unconjugated forms of Atg8 can be separated by standard SDS-PAGE and urea-containing SDS-PAGE in mammals [29] and yeast [15], respectively. Although Atg8 gains molecular mass when conjugated to PE, its apparent mobility in SDS-PAGE increases probably because of the highly hydrophobic nature of PE. However, PfAtg8 was detected only as a single band in SDS-PAGE (Figure 2A), and no extra band was identified even in the presence of 6 M urea (data not shown). This suggests that the majority of PfAtg8 is present in either the conjugated or unconjugated form. When PfAtg8 was expressed in mammalian cells, PfAtg8 was also detected as a single band, although the mobility was lower than that of PfAtg8 expressed in parasites (Figure 2C). As it is unlikely that PfAtg8 conjugates with PE in mammalian cells, the band detected in mammalian cells is likely to represent the mobility of the unconjugated form. We therefore speculate that PfAtg8 is present primarily in a PE-conjugated form in *Plasmodium*.

We thus investigated whether or not PfAtg8 is membrane bound. The lysates of asynchronous parasites were fractionated by differential centrifugation. PfAtg8 was mainly collected in a low-speed (13,000×*g*) pellet (LSP) fraction (Figure 2D). PfAtg8 in the LSP fraction could be solubilized by treatment with 2% Triton X-100, but not with 2 M urea (Figure 2E). This behavior is characteristic of integral membrane proteins, and Atg8 – PE of other organisms have been known to behave in this manner [15], [29]. Thus our data suggest that most PfAtg8 is membrane-associated in intra-erythrocytic *Plasmodium*.

In yeast and mammalian cells, Atg8/LC3 associating with the inner autophagosomal membrane is degraded upon fusion with lysosomes [31]. Chloroquine, a well-known anti-malarial drug, impairs lysosomal acidification, and thereby blocks degradation of LC3 in the lysosome [31]. If PfAtg8 is attached to the autophagosomal membrane in *P. falciparum* as are Atg8 or LC3 in yeasts and mammals, this protein should also be degraded within the lytic organelles such as the food vacuole. Chloroquine may affect this process and therefore we examined its effect on the level of PfAtg8. However, treatment of the parasite growing in erythrocytes with chloroquine did not increase the amount of PfAtg8 (Figure 2F), suggesting that PfAtg8 is not involved in the autophagic process at this stage of the parasite's life cycle.

### PfAtg8 localizes to the apicoplast membrane

**Figure 3:**
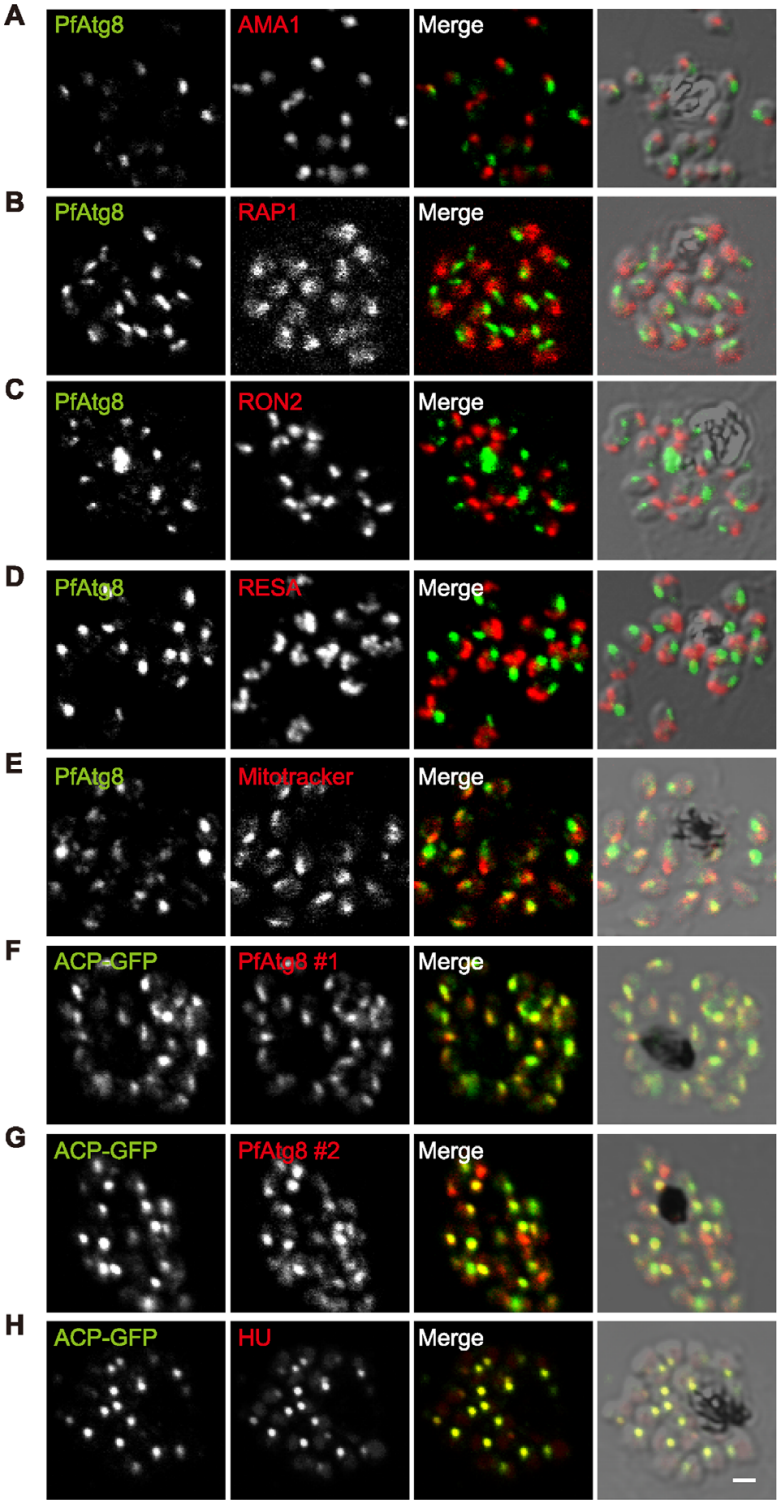
PfAtg8 localizes to the apicoplast. *P. falciparum* FCR3 (A–E) and *P. falciparum* 3D7 transfected with ACP-GFP (F–H) were stained with the indicated organelle markers and visualized by confocal microscopy (because ACP-GFP was not uniformly expressed, some merozoites displayed only faint GFP signals). Anti-PfAtg8 antibody #1 was used in (A–F), and anti-PfAtg8 antibody #2 was used in (G). Apical membrane antigen 1 (AMA1) as a microneme marker (A), rhoptry-associated protein 1 (RAP1) as a rhoptry body marker (B), rhoptry neck protein 2 (RON2) as a rhoptry neck marker (C), the ring-infected erythrocyte surface antigen (RESA) as a dense granule marker (D), MitoTrackerRed CMXRos as a mitochondria marker (E), ACP-GFP (F–H) and the organellar histone-like protein PfHU (H) as an apicoplast marker were used. Scale bar, 1 μm.

We next determined the subcellular localization of PfAtg8 by immunofluorescence microscopy. In segmented schizonts, the PfAtg8 signal was detected as a single punctate structure in each merozoite (Figure 3). The size of each punctate structure was approximately 200–400 nm. The fact that every parasite possessed one such structure precluded the possibility that the PfAtg8-positive structure is rapidly turned over in the same way as the autophagosome.

**Figure 4:**
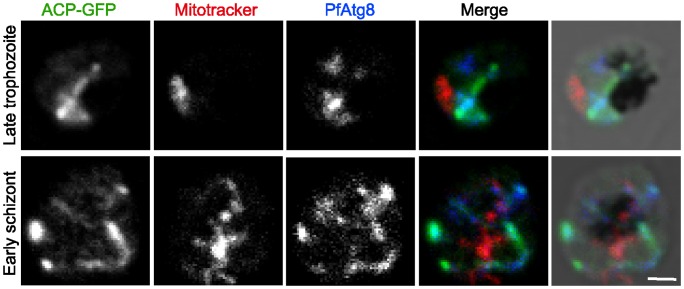
PfAtg8 localizes to tubular and branched apicoplasts. *P. falciparum* transfectant expressing ACP-GFP at late trophozoite and early schizont stages was stained with anti-GFP and anti-PfAtg8 antibodies and MitoTrackerRed CMXRos, and visualized by confocal microscopy. Scale bar, 1 μm.

The nature of these PfAtg8-positive structures was further characterized by double staining with organelle markers. PfAtg8 did not colocalize with any markers for the merozoite apical organelles such as the microneme (Figure 3A), rhoptry body (Figure 3B), rhoptry neck (Figure 3C), and dense granule (Figure 3D). By contrast, PfAtg8 colocalized with the apicoplast-localizing green fluorescent protein (ACP-GFP) (some of the parasites displayed only weak ACP-GFP expression) [32], [33] (Figure 3 F). We confirmed the colocalization between ACP-GFP and PfAtg8 using the independent anti-PfAtg8 antibody #2 (Figure 3G). All the organelles labeled with the ACP-GFP antibody also reacted with the antibody against the plastid-localizing PfHU (histone-like protein, heat unstable), an endogenous apicoplast marker (Figure 3H) [34]. The PfAtg8-positive structure was observed in close proximity to the mitochondrion, and even appeared to overlap part of the organelle (Figure 3E). This partial overlapping between mitochondria and PfAtg8 is consistent with the fact that the apicoplast and the mitochondrion are juxtaposed, probably maintaining physical contact, in the parasite cell [35], [36]. To better dissect the localization of PfAtg8, we looked at the parasites at an earlier stage. Morphology of the apicoplast dramatically changes during development inside erythrocytes [32], [37]. At the late trophozoite to early schizont stages, the apicoplast forms a tubular or branched shape. In fact, PfAtg8 localized to the tubular or branched apicoplasts, which were clearly distinct from the mitochondria (Figure 4). Taken together, these data suggest that PfAtg8 localizes to the apicoplast during normal development.

**Figure 5:**
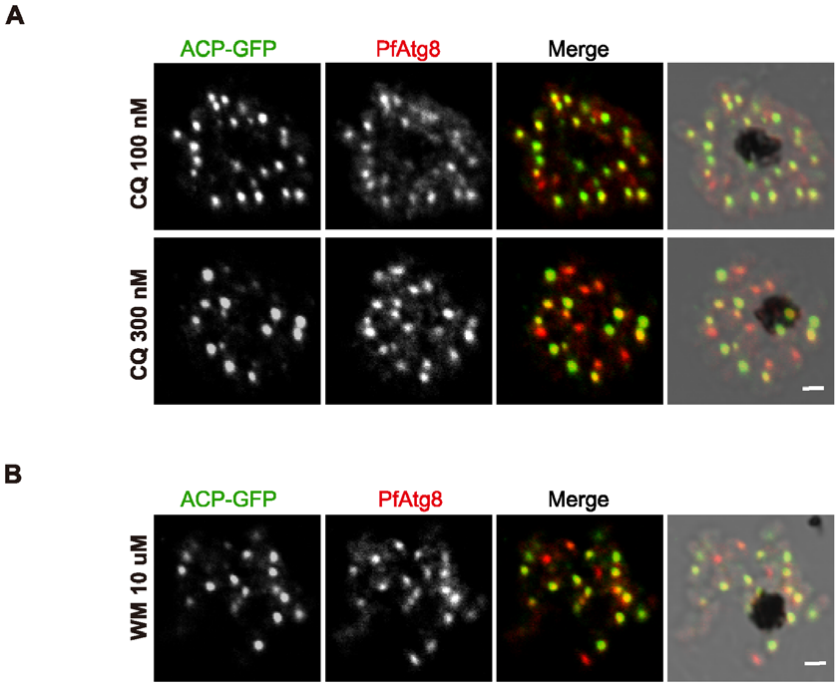
PfAtg8 localization is not affected by chloroquine or wortmannin treatment. *P. falciparum* transfectant expressing ACP-GFP was treated with chloroquine (100 or 300 nM) (A), or wortmannin (10 μM) (B) for 2 h. Scale bar, 1 μm.

Even though the main localization of PfAtg8 is the apicoplast, PfAtg8 could be present on other structures such as autophagosomes. However, the localization pattern of PfAtg8 was not significantly changed by treatment of chloroquine (Figure 5A), which can typically accumulate autophagosomes/autolysosomes in mammalian cells (as we mentioned above, ACP-GFP was not uniformly expressed and some merozoites displayed only faint GFP signals) [38]. Thus, we could not conclude whether *P. falciparum* can generate PfAtg8-positive autophagosomes. Furthermore, treatment with wortmannin, a PtdIns 3-kinase inhibitor, did not affect PfAtg8 localization, suggesting that association of PfAtg8 with the apicoplast membrane is independent of PtdIns 3-phosphate (Figure 5B).

**Figure 6:**
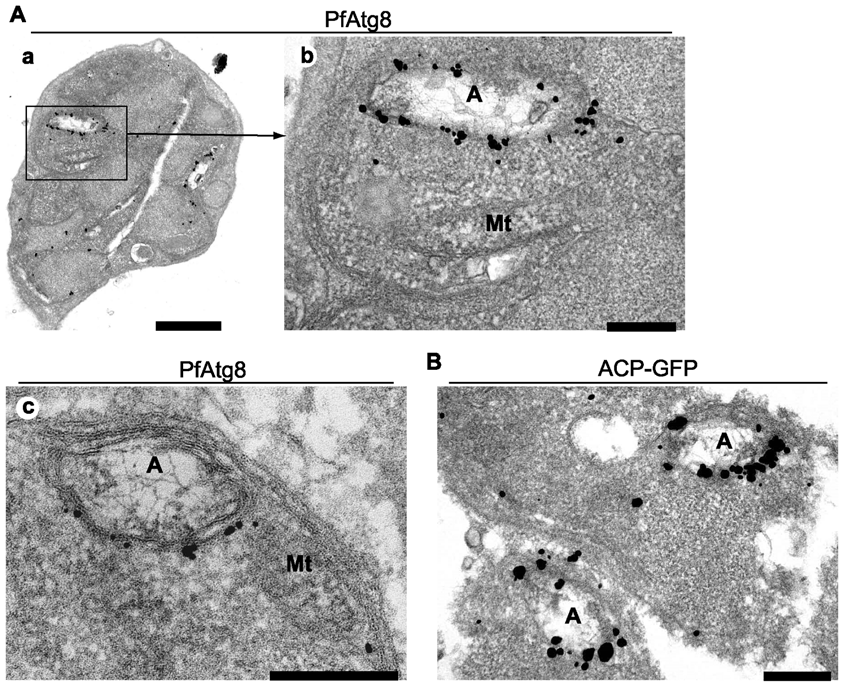
PfAtg8 is associated with the apicoplast membrane. (A) *P. falciparum* FCR3 parasites at the schizont stage were analyzed by immunoelectron microscopy (immunogold and silver enhancement method) with an antibody against PfAtg8 (#1). (a) A schizont in an erythrocyte. (b) A magnified image of the area indicated in (a). (c) Another typical image of a PfAtg8-positive structure. (B) *P. falciparum* transfectant expressing ACP-GFP was analyzed as in panel (A) with an antibody against GFP. A, apicoplast; Mt, mitochondrion. Scale bars, (A, a) 1 μm, (A, b and c, and B) 200 nm.

We further analyzed the localization of PfAtg8 by immunoelectron microscopy using the anti-PfAtg8 antibody. The silver-enhanced gold particles specifically associated with multiple membrane-bound organelles (Figure 6A). The inside of the organelles were filled with relatively low-density materials and a fiber-like structure, which are features of the apicoplast [35], [39], [40]. These characteristics suggest that the organelles surrounded by PfAtg8 were different from autophagosomes, which are defined as double membrane-bound organelles containing undigested cytoplasmic materials, and from autolysosomes, which contain degraded materials [41]. We did not observe such autophagic structures in the parasites in the erythrocytic stage. To confirm that the PfAtg8-positive multi-membrane structures were indeed apicoplasts, we performed immunoelectron microscopy of ACP-GFP-expressing parasites. Anti-GFP antibody specifically reacted with multi-membrane organelles that looked the same as the structures to which PfAtg8 localized (Figure 6A and 6B). The PfAtg8 signals were not detected on the mitochondrion that was in close proximity to the apicoplast (Figure 6A). This suggests that the fluorescence signal detected apparently in the mitochondrion (Figure 3E) was caused by the spatial overlap of the organelle and the apicoplast. Taken together, these data suggest that PfAtg8 specifically localizes to the membrane of the apicoplast in *P. falciparum*.

## Discussion

We report here that PfAtg8 is associated with the apicoplast, probably with the outermost membrane likely in a lipid-conjugated form during the erythrocytic stage. Although we did not detect any autophagosome-like structures in *P. falciparum* even under chloroquine treatment, we do not rule out the possibility that Atg8 can localize to autophagosomes if such structures are formed in *Plasmodium*. The apicoplast is a non-photosynthetic plastid, which is an essential organelle possessing its own genome [40], [42], [43]. The apicoplast is involved in several metabolic pathways such as biosynthesis of fatty acids, haem, isoprenoid (required for tRNA modification, etc) and iron–sulphur clusters. *P. falciparum* has one plastid, and it physically contacts with the mitochondrion during intraerythrocyte stage [35], [36], although the relationship between these two organelles seem to be more complicated in liver stages [44]. The apicoplast has four membranes and that is believed to explain the origin of the organelle from a secondary endosymbiotic alga, most likely a red alga [43], [45], [46]. The outer two membranes appear to be related to the ER. Nuclear-encoded apicoplast proteins possess an N-terminal signal peptide that is essential for their delivery to the apicoplast via Golgi-independent transport [32], [40], [47], [48], [49]. ER-associated protein degradation-like machinery exists in the second outermost membrane [50], [51]. Although the autophagosome does not have any ER-related proteins and contribution of other organelles such as mitochondria [52] and the plasma membrane [53], autophagosomes are basically generated on or in close proximity to the ER [8], [54]; even direct membrane continuity between the ER and autophagosome has been suggested [55], [56]. Therefore, Atg8 may have a shared role in biogenesis of ER-related organelles.

To date, no particular contribution of PfAtg8 on the apicoplast or its biogenesis has been predicted. This protein was suggested to be essential because deletion of the *Atg8* gene in *P. berghei* causes a lethal phenotype [13], [27]. Our ultrastructural analysis, as well as the fact that the parasite has one apicoplast throughout its cell cycle (except for a short period of organellar division), suggest that PfAtg8 is unlikely to be used for autophagic degradation of the apicoplast. Conditional targeting of *Atg3* in *T. gondii* resulted in a severe growth defect with altered mitochondrial morphology [26]. The observed mitochondrial defects might have been caused by a defect in mitochondrial autophagy (mitophagy) as suggested by Besteiro et al., but it may be due to an impaired apicoplast–mitochondria relationship. If the Atg8 conjugation system is involved in essential cellular activities of the malaria parasites, compounds that can inhibit the conjugation reaction (i.e. inhibitors of PfAtg7 or PfAtg3) would be promising therapeutic tools.

*P. falciparum* contains the class III PtdIns 3-kinase Vps34 (Figure 1A) and PtdIns 3-phosphate is present on both the food vacuole and the apiocoplast membrane [30]. As the Atg8 system functions downstream of the PtdIns 3-kinase complex in starvation-induced autophagy in both yeast and mammals [9], [10], we speculated that association between PfAtg8 and the apicoplast membrane could depend on PtdIns 3-kinase activity. However, we observed that treatment of *P. falciparum* with wortmannin did not affect the localization of PfAtg8 (Figure 5B). Recently, it was reported that LC3 (a mammalian Atg8) can associate with membranes even in the absence of upstream Atg factors such as the ULK1/Atg1 complex, Atg9, and PtdIns 3-kinase activity in some types of selective autophagy such as xenophagy against *Salmonella* [57] and Parkin-mediated mitophagy [58]. Nonetheless, the membrane association with LC3 still depends on the two ubiquitin-like LC3 and Atg12 conjugation systems. Therefore, it is possible that the PfAtg8 conjugation system has a unique function, which is independent of most other Atg proteins.

Another interesting issue is the requirement of the Atg12 conjugation system. In the autophagy pathway, the Atg8/LC3 conjugation reaction requires an E3-like activity of the Atg12–Atg5 conjugate both in yeast and mammals [59], [60]. However, PfAtg12 lacks the C-terminal glycine residue (Figure S2), which is essential for formation of an isopeptide bond with Atg5. An attractive hypothesis is that PfAtg12 alone may have E3-like activity without Atg5, although the *P. falciparum* genome contains a gene encoding a potential Atg5 homolog (Figure S1). The gene, *PF14_0283* encodes an 863-amino acid (aa) protein that is much larger than yeast (294 aa) and human Atg5 (275 aa). Because of the presence of a number of insertion sequences, it is important to examine whether this Atg5 candidate is a functional Atg5 ortholog that should conjugate and collaborate with PfAtg12 in the organism. Further understanding the roles of Atg8 and Atg12 in *Plasmodium* will provide a general insight into the functions of Atg proteins even in the autophagy pathway.

## Materials and Methods

### Parasite culture

*P. falciparum* strain FCR3 was cultured in human B^+^ erythrocytes as described [61]. In some subcellular localization experiments, the 3D7 parasite strain transfected with pSSPF2/GFP-ACP was used; the transfectant was cultured in the standard culture medium supplemented with 5 nM WR99210 [33]. Where indicated, chloroquine (Sigma-Aldrich) was added to the culture medium. For synchronizing the culture, the red blood cells infected by the late stage schizont were recovered from asynchronous culture by 60% Percoll (GE healthcare) density centrifugation at 2000×*g* for 20 min. After 4 h the culture was treated with 5% D-sorbitol [62], yielding parasites tightly synchronized in the early ring stage (0–4 h after parasite invasion of the erythrocyte).

### Cloning of PfAtg8 cDNA and generation of anti-PfAtg8 antibodies

RNA extraction and cDNA synthesis were carried out as described previously [63], [64]. GST-fused PfAtg8 recombinant protein was generated using a wheat germ cell-free system [65]. Two independent anti-PfAtg8 antisera (#1 and #2) were raised in two New Zealand white rabbits and the antibodies were purified using GST-PfAtg8 recombinant protein. Animal experimental protocols were approved by the Institutional Animal Care and Use Committee of Tokyo Medical and Dental University (No. 0110115A).

### Immunoblotting

Parasites were collected from erythrocytes by treatment with 0.15% saponin (Sigma) in phosphate-buffered saline (PBS) with Complete Protease Inhibitor cocktail (Roche Applied Science), washed three times in PBS and lysed in sample buffer. Parasite extracts were loaded onto 13.5% SDS gel and transferred to a PVDF membrane. Blots were blocked with 5% skim milk in Tris-buffered saline with 0.01% Tween 20 (TBST) and stained with primary antibodies overnight at 4°C. The following primary antibodies were used: rabbit anti-PfAtg8 and mouse monoclonal anti-PfHSP70 antibodies (1∶100) [66], [67]. After washing with TBST, blots were stained with HRP-conjugated secondary antibodies and visualized with SuperSignal West Pico Chemiluminescent substrate (Thermo Fisher Scientific).

### Subcellular fractionation

Asynchronous parasites were harvested as described above. Parasite pellets were disrupted by three cycles of freezing/thawing in MSE buffer (225 mM mannitol, 75 mM sucrose, 0.1 mM EDTA, and 3 mM Tris-HCl [pH 7.4]). Cell debris and intact erythrocytes were removed by centrifugation at 800×*g* for 5 min. The supernatant was spun at 13,000×*g* for 15 min to separate the LSP, and the supernatant was centrifuged again at 100,000×*g* for 60 min to generate the high-speed pellet (HSP) and high-speed supernatant (HSS). The LSP and HSP were resuspended in the same buffer. To analyze solubility, each sample was incubated with 2 M urea or 2% Triton X-100 on ice for 1 h, and then centrifuged at 100,000×*g* for 1 h. The samples were precipitated with ice-cold acetone, resuspended in SDS-PAGE sample buffer, and analyzed by SDS-PAGE.

### Expression of PfAtg8 in mammalian cells

PfAtg8 cDNA was inserted into a pCI-neo mammalian expression plasmid (Promega) and transfected into HEK293T cells [68] using Lipofectamine 2000 reagent (Invitrogen). Total cell lysates were subjected to SDS-PAGE and immunoblot analysis.

### Immunofluorescence microscopy

Parasite thin blood smears were fixed with 4% paraformaldehyde/PBS for 10 min and samples were permealized with 0.1% Triton X-100/PBS for 15 min. After blocking with 3% bovine serum albumin/PBS for 1 h, samples were incubated with primary and secondary antibodies for 2 h and 1 h, respectively. The smears were mounted with Prolong Gold (Invitrogen). All reactions were carried out at room temperature. Samples were observed with a confocal laser microscope (FV1000D IX81, Olympus) using a 60x PlanApoN oil immersion lens (1.42 NA; Olympus). The following primary antibodies were used: purified rabbit anti-PfAtg8 (1∶200 for #1, and 1∶100 for #2) antibody, mouse anti-apical membrane antigen (AMA)1 (1∶500) [67], anti-rhoptry-associated protein 1 (RAP1, 1∶200) [67], anti-rhoptry neck protein 2 (RON2, 1∶200) [69], and anti-ring-infected erythrocyte surface antigen (RESA) (23/9, 1∶200) [70] antibodies, rabbit anti- PfHU (organellar histone-like protein) antibody [34], and rat anti-GFP antibody (Nacalai Tesque). For visualizing the mitochondrion, parasites were preincubated for 30 min with complete culture medium containing 100 nM MitoTracker Red CMXRos (Molecular Probes).

### Immunoelectron microscopy

Mature schizont stage parasites were enriched from synchronous culture using MACS 25LD columns (MiltenyiBiotec) as previously described [71]. For immunoelectron microscopy of *P. falciparum*, the previously described pre-embedding silver enhancement immunogold method [72] was used with slight modifications. The parasitized erythrocytes were fixed in 4% paraformaldehyde and 0.0075% glutaraldehyde dissolved in 0.1 M sodium phosphate buffer (PB) (pH 7.4) for 2 h and then washed three times with PB. Then the cells were permeabilized in liquid nitrogen and incubated in a blocking buffer containing 0.005% saponin, 10% goat serum, 0.1% cold water fish gelatin, and 10% bovine serum albumin for 30 min, and reacted with rabbit anti-PfAtg8 (#1) or rat monoclonal anti-GFP (IgG2a, Nacalai Tesque #04404-84) in blocking buffer at 4°C overnight. Next the cells were washed in PB containing 0.005% saponin and incubated with goat anti-rabbit IgG or anti-rat IgG conjugated with colloidal gold (1.4-nm diameter, Nanogold, Nanoprobes) in blocking buffer for 2 h at room temperature. Cells were washed five times with PB containing 0.005% saponin for 10 min, washed with PB for 5 min, and fixed with 1% glutaraldehyde for 10 min. After washing, the gold partiles were intensified using a silver enhancement kit (HQ silver, Nanoprobes) for 6 min at 20°C in the dark. After washing in distilled water, the cells were post-fixed with 0.03% OsO_4_ for 15 min at 4°C. After washing with PB, cells were resuspended in 2% gelatin (Sigma) and pelleted again. Microcentrifuge tubes were plunged into ice-cold water to quickly solidify the gelatin with the cells. The tip of the tube was cut open and the cell pellets were retrieved into 15% ethanol, and cut into 1-mm^3^ blocks. The blocks were suspended and dehydrated with a graded series of ethanol concentrations, and embedded in epoxy resin. Ultrathin sections were doubly stained with uranyl acetate and lead citrate and observed using a Hitachi H7100 electron microscope.

## Supporting Information

Figure S1 **Sequence alignment of Atg5 homologs.** Alignment of the sequences of *S. cerevisiae* Atg5, *H. sapiens* Atg5 and *P. falciparum* Atg5. Asterisk (*) shows the position of the Lys residue that receives Atg12 conjugation in yeast and human. This Lys is conserved in PfAtg5.
(TIF)

Figure S2 **Sequence alignment of Atg12 homologs.** Alignment of the sequences of *S. cerevisiae* Atg12, *H. sapiens* Atg12 and *P. falciparum* Atg12. Asterisk (*) shows the C-terminal Gly residue essential for conjugation with Atg5 in yeast and human. PfAtg12 lacks this Gly residue.
(TIF)

Figure S3 **Sequence alignment of Atg18 homologs.** Alignment of the sequences of *S. cerevisiae* Atg18, *H. sapiens* WIPI1 and *P. falciparum* Atg18. Asterisk (*) shows the motif required for PtdIns 3-phosphate binding.
(TIF)
