# Supplementary figures and images for: Autophagy-Related Atg8 Localizes to the Apicoplast of the Human Malaria Parasite Plasmodium falciparum
Source: PLoS One. 2012 Aug 10;7(8):e42977. doi: 10.1371/journal.pone.0042977 (PMC3416769; doi:10.1371/journal.pone.0042977)

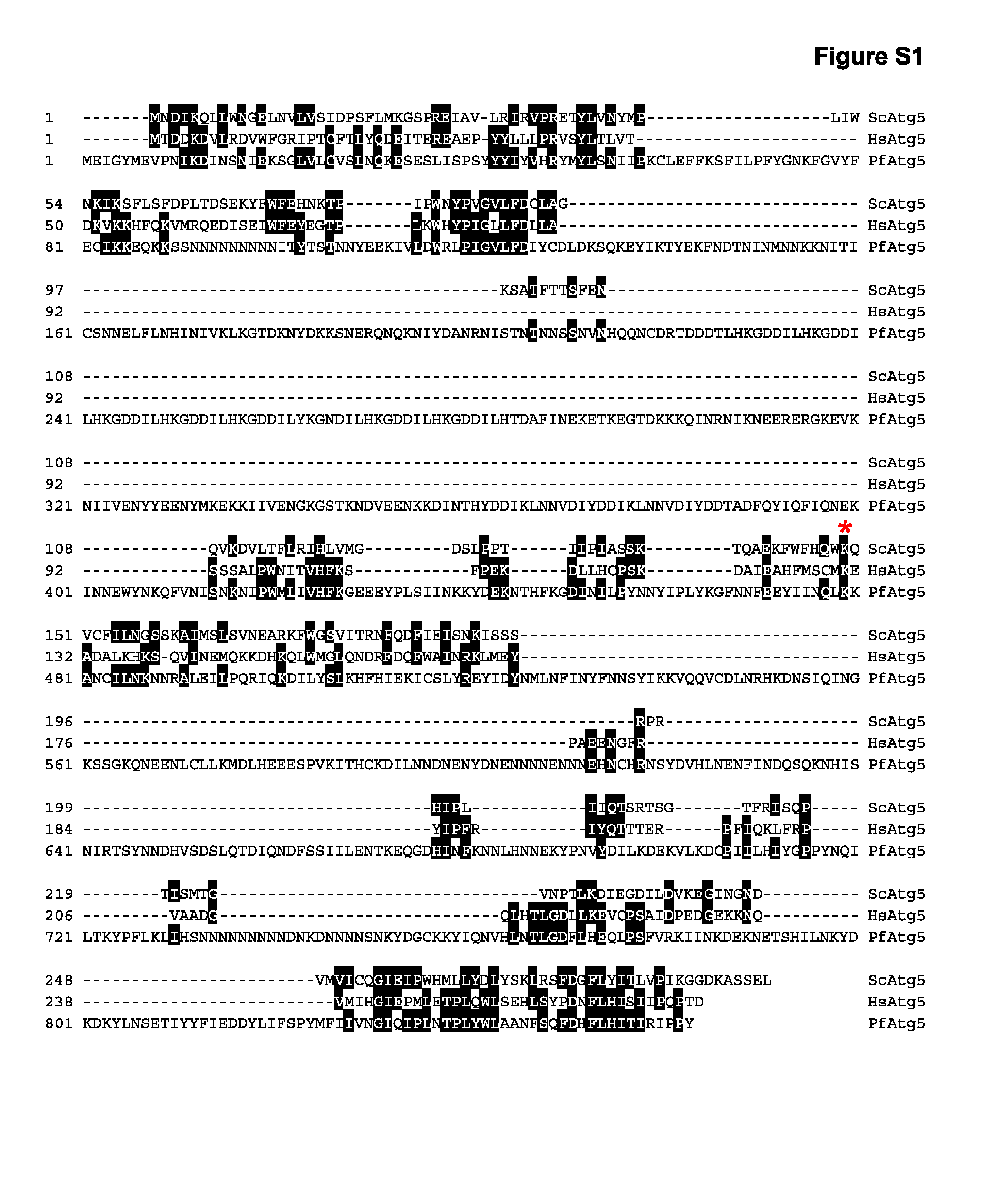

Supplement: Figure S1 — Sequence alignment of Atg5 homologs. Alignment of the sequences of S. cerevisiae Atg5, H. sapiens Atg5 and P. falciparum Atg5. Asterisk (*) shows the position of the Lys residue that receives Atg12 conjugation in yeast and human. This Lys is conserved in PfAtg5. (TIF) [file pone.0042977.s001.tif]

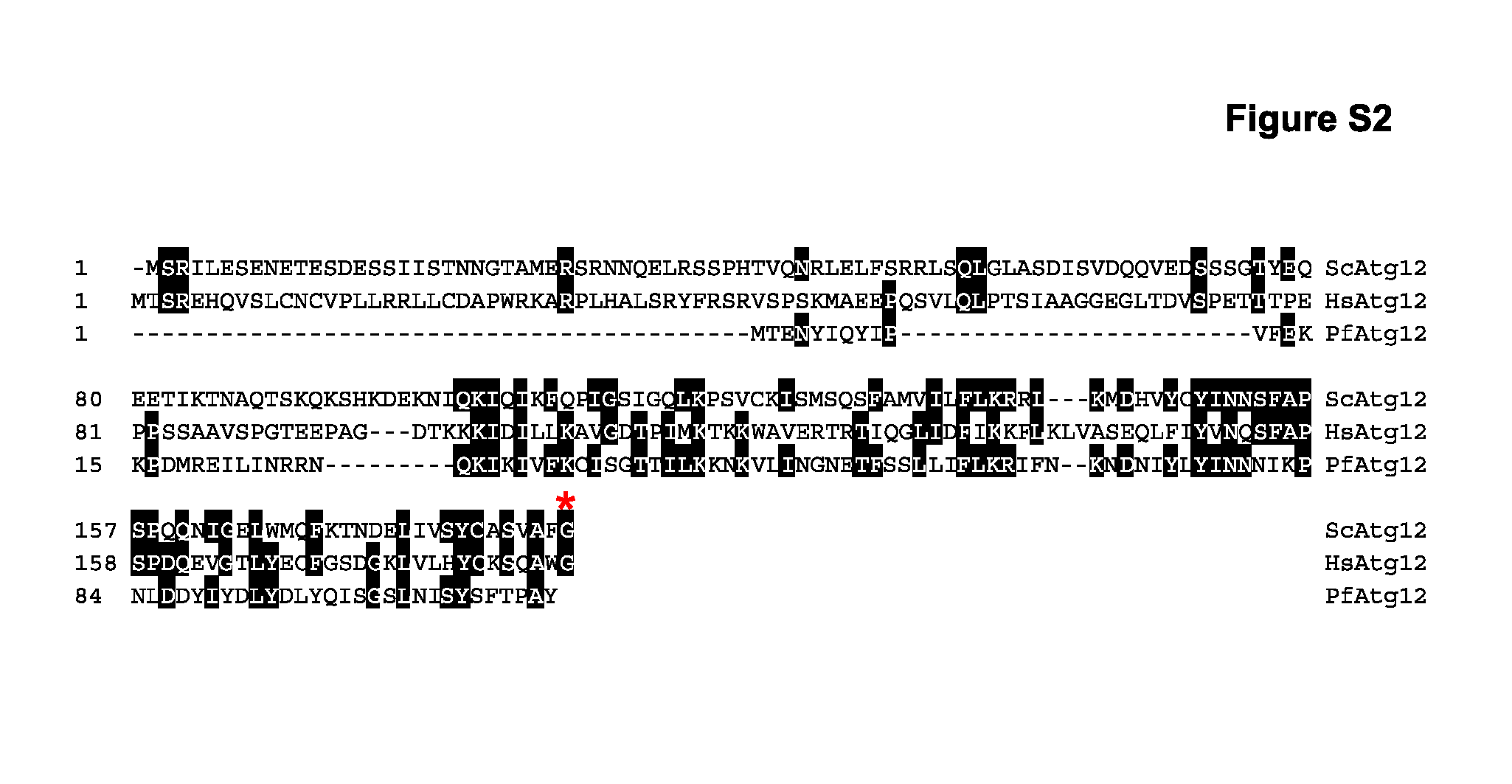

Supplement: Figure S2 — Sequence alignment of Atg12 homologs. Alignment of the sequences of S. cerevisiae Atg12, H. sapiens Atg12 and P. falciparum Atg12. Asterisk (*) shows the C-terminal Gly residue essential for conjugation with Atg5 in yeast and human. PfAtg12 lacks this Gly residue. (TIF) [file pone.0042977.s002.tif]

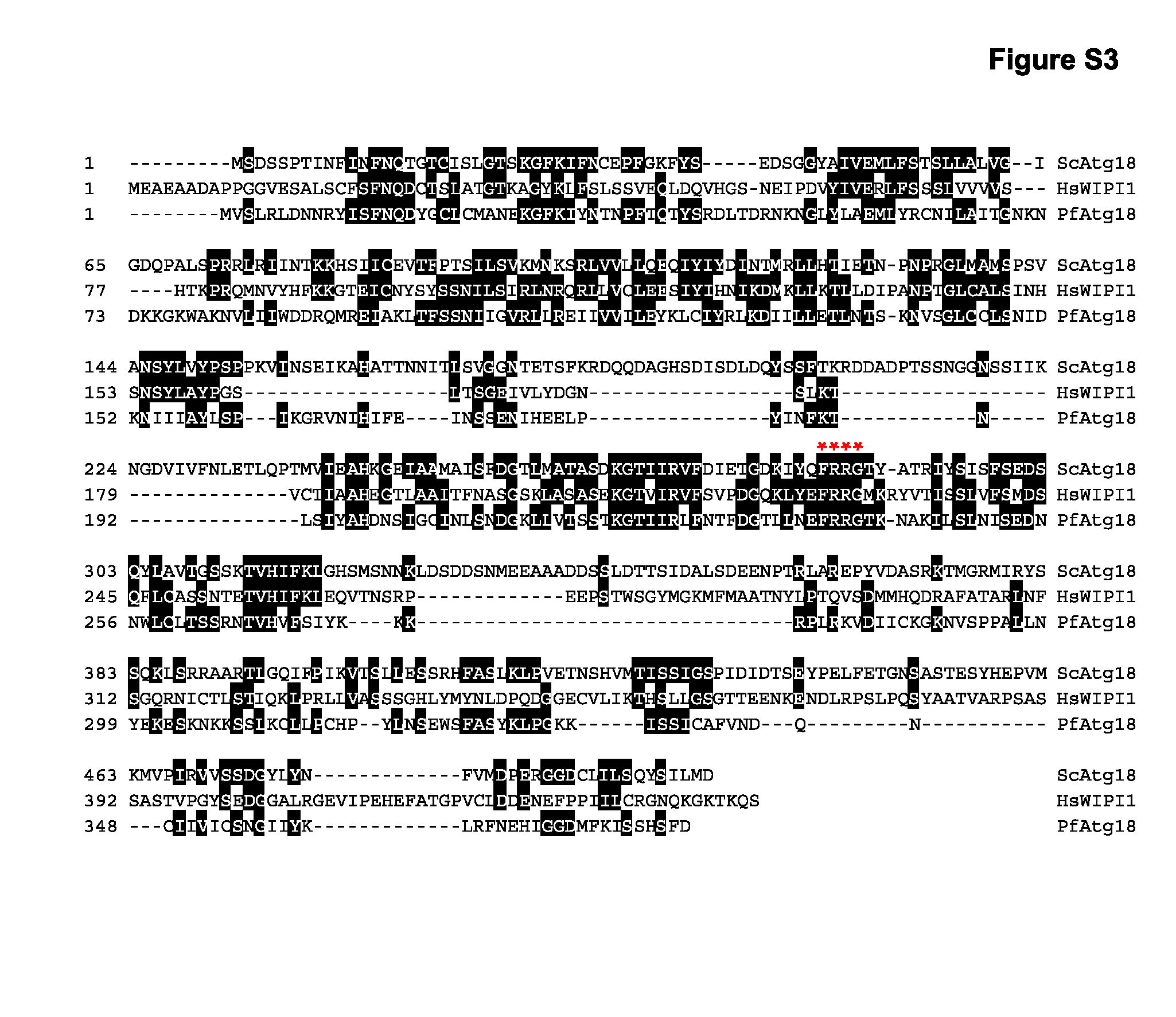

Supplement: Figure S3 — Sequence alignment of Atg18 homologs. Alignment of the sequences of S. cerevisiae Atg18, H. sapiens WIPI1 and P. falciparum Atg18. Asterisk (*) shows the motif required for PtdIns 3-phosphate binding. (TIF) [file pone.0042977.s003.tif]
